# Computational study on catalyst-free BCl_3_-promoted chloroboration of carbonyl compounds†

**DOI:** 10.1039/d4ra06893a

**Published:** 2025-01-28

**Authors:** Abera Tadie Derese, Mengistu Gemech Menkir, Mihret Kendie Wolie, Desalegn Addis Yemam

**Affiliations:** a Department of Chemistry, Debre Tabor University Ethiopia aberatadie@gmail.com; b Department of Chemistry, Bahir Dar University Ethiopia; c Department of Chemistry, Debark University Ethiopia; d Department of Chemistry, Debre Tabor University Ethiopia

## Abstract

DFT calculations were performed to investigate the possible reaction mechanisms underlying catalyst-free chloroboration reactions of carbonyl compounds with BCl_3_. The interaction between BCl_3_ and the C=O moiety of carbonyl compounds is a two-step reaction. In the first step, B of BCl_3_ forms a bond with the O of the C=O moiety, followed by the 1,3-Cl migration process from BCl_3_ to the C of the carbonyl group. To indicate the versatility of our synthetic methodology, a catalyst-free chloroboration of a variety of aldehydes and ketones with a broad range of electron-donating and electron-withdrawing groups with BCl_3_ was checked. According to DFT results, BCl_3_-induced chloroboration of aldehydes and ketones progressed under a kinetically favorable condition with <20 kcal mol^−1^ of activation free energy.

## Abstract 2

DFT calculations were performed to investigate the possible reaction mechanisms underlying catalyst-free chloroboration reactions of carbonyl compounds with BCl_3_.

## Graphical abstract

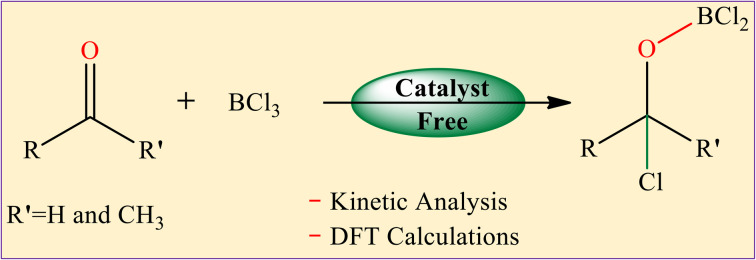

## Introduction

Boronate esters, which are produced when carbonyl compounds undergo hydroboration, are frequently employed as significant applications in biomedical and pharmaceutical research, as well as in organic synthesis.^1–8^ Because boronate esters are important as synthetic intermediates in a wide range of chemical processes, several techniques for producing them have been devised. Traditionally, stoichiometric additions of reactive BH_3_ to generate borates, which can be hydrolyzed into alcohols, or stoichiometric amounts of dangerous metal-hydrides, such as LiAlH_4_ and NaBH_4_, have been used to induce hydroboration of C=O bonds.^9–14^ In terms of cost and atom efficiency, carbonyl reduction using transition metal-catalyzed hydrogenation procedures employing combustible and highly pressured H_2_ gas is also optimal.^15,16^ Regardless of their amazing success, these procedures exhibit low yields, poor functional group compatibility, and the need for stoichiometric reagents for large-scale applications.

The discovery of transition metal-catalyzed hydroboration, which uses pinacolborane (HBpin) or catecholborane (HBcat) to selectively reduce unsaturated carbonyl compounds, has been a significant advancement in the past years.^17–22^ Classically, different methods with the involvement of metals, either as catalysts or in stoichiometric proportions, have been discovered to synthesize organoboron compounds.^23–26^ However, the involvement of metals may render these preparative techniques less economical and environmentally benign, and it may result in heavy metal contamination of the final boron products.^27^

To eliminate the above-listed drawbacks, transition-metal-free catalytic methods were widely adopted over the traditional approach.^28^ Among these, transition-metal-free catalytic diboration of unsaturated hydrocarbons,^29,30^ transition-metal-free catalytic β-boration of α,β-unsaturated compounds,^31^ transition-metal-free catalytic borylation of allylic and propargylic alcohols,^32^ and transition-metal-free catalytic hydroboration of unsaturated hydrocarbons^33–36^ have been reported.

The use of protocols in organic transformations that do not require solvents or catalysts has gained considerable interest lately as a result of the growing need for low-cost, atom-economical, and environmentally safe synthetic processes.^37,38^ Recently, catalyst-free and solvent-free hydroboration of carbonyl compounds using pinacol borane has been reported. For example, in 2018, Stachowiak and coworkers studied catalyst-free and solvent-free hydroboration of aldehydes.^39^ In addition, Wang and colleagues experimentally and computationally investigated catalyst-free and solvent-free hydroboration of ketones^40^ and carboxylic acids.^41^ However, for the hydroboration of ketones and carboxylic acids, computational results reveal that the reactions are hard to proceed at room temperature because of the high energy barrier.

Furthermore, borylative cyclization of unsaturated compounds without any catalyst, such as intramolecular amination of alkenes and alkynes, metal-free borylative cyclization of alkynes, intramolecular aminoboration of allenes, and catalyst-free annulative thioboration of unfunctionalized olefins was demonstrated using BCl_3_ as a boron source.^42–45^ However, the action of BCl_3_ on catalyst-free chloroboration of carbonyl compounds has not yet studied. Inspired by the work described above, we decided to carry computational work on BCl_3_-induced chloroboration of carbonyl compounds in a catalyst-free manner. In this study, we performed a DFT calculation for catalyst-free chloroboration of aldehydes and ketones using BCl_3_ as a boron source.

### Computational methodology

The Gaussian 09 ^46^ computational program suite, along with its Gauss View 5.0 graphical user interface, was utilized for all calculations carried out in this work. Using the 6-31+G(d) basis set and M062X^47^ hybrid functional, the geometry optimizations of all the structures were performed. Frequency calculations were performed at the same theoretical level for each stationary point to classify them as minima (no imaginary frequency) or transition states (one imaginary frequency) and to derive thermodynamic energy corrections. To confirm that the transition states link two pertinent local minima along the potential energy surface, intrinsic reaction coordinate (IRC)^48^ computations were performed on the transition structures. To obtain more accurate energy estimates, the larger basis set 6-311++G(d,p) and M062X^49^ functional were used in single-point energy calculations in toluene based on gas phase optimized geometries utilizing the PCM^50^ model.

## Results and discussion

**Scheme 1:**
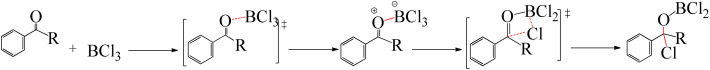
Proposed mechanism for the BCl_3_-mediated chloroboration of aldehydes and ketones (R=H for aldehydes; R=CH_3_ for ketones).

**Fig. 1:**
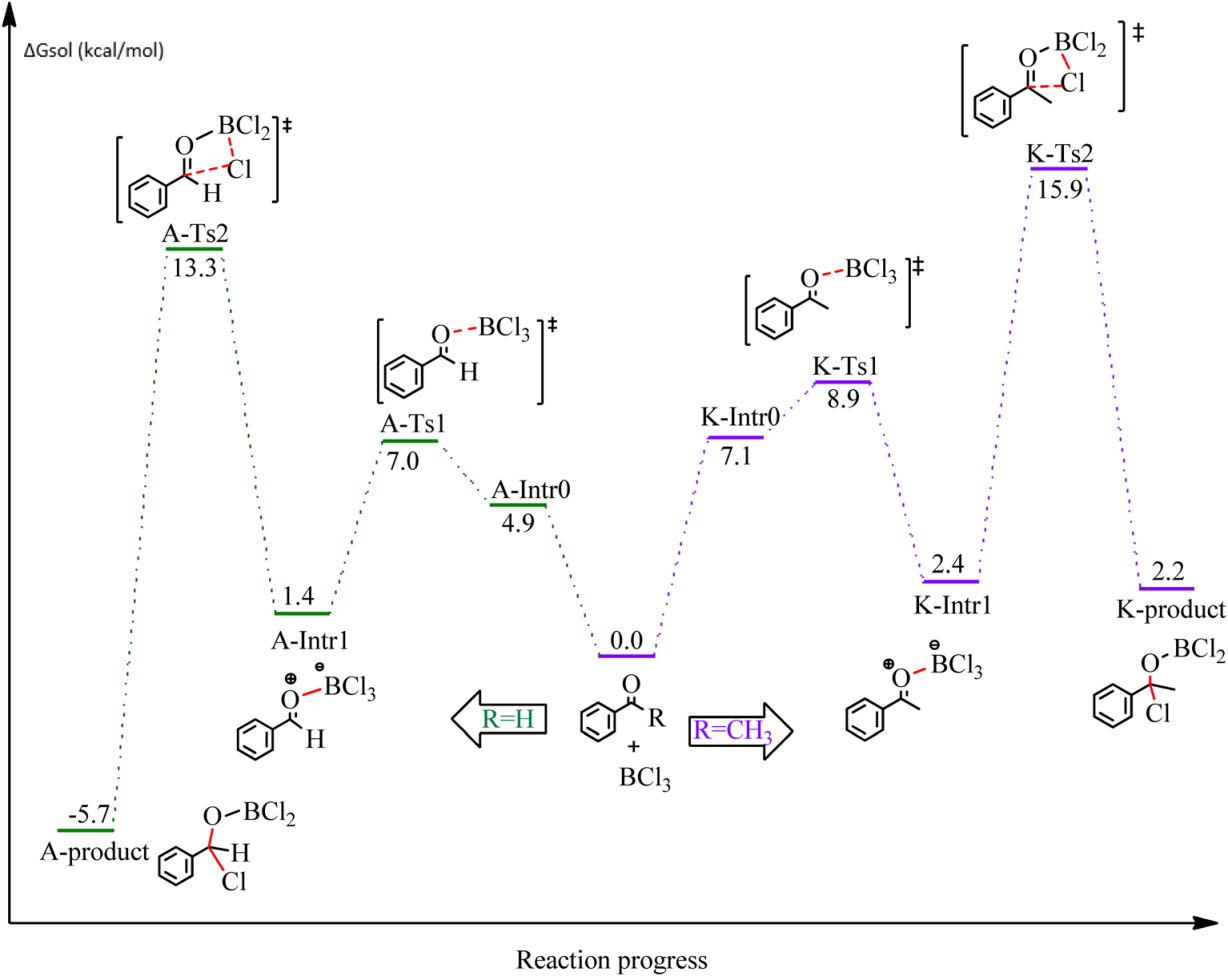
DFT-computed free energies for the chloroboration reaction of benzaldehyde and acetophenone.

First, the mechanism of benzaldehyde and acetophenone and the substrates of the chloroboration model was investigated. Scheme 1 depicts the proposed reaction mechanism for BCl_3_-promoted chloroboration of aldehydes and ketones to produce boronate esters. The first and most common step for both carbonyl substrates is the bond formation between the B of BCl_3_ and O of the C=O moiety. The second step involves a chloride shift from BCl_3_ to the carbonyl carbon to afford a chlorinated borane. DFT calculations were performed to investigate the detailed proposed mechanism. The calculated energetic profiles are shown in Fig. 1.

We started the study by reacting benzaldehyde and BCl_3_ without the use of a catalyst. Based on the DFT calculation results shown in Fig. 1, the production of the coordinated complex A-Intr0 between the C=O group of the model reactant (benzaldehyde) and BCl_3_ is found to be 4.9 kcal mol^−1^. Through A-Ts1, A-Intr0 is subsequently transformed into the zwitterion intermediate A-Intr1 at a lower activation energy of 2.1 kcal mol^−1^. The generation of the borylated intermediate A-product *via* the four-membered ring transition state A-Ts2 requires 11.9 kcal mol^−1^, which corresponds to the formation of C–Cl. The breaking B–Cl distance is 2.12 Å, whereas the forming C–Cl distance is 2.18 Å.

Following the identification of the most advantageous route for the chloroboration of the aldehyde model reactant, we perform analogous DFT computations on ketones using acetophenone as a substrate. The complexation process between BCl_3_ and the C=O moiety of the model reactant, acetophenone, initiates the reaction. Fig. 1 illustrates that the former transition state, K-Ts1, has a lower energy barrier of 1.8 kcal mol^−1^, making it more kinetically beneficial to afford K-Intr1. Following the formation of K-Intr1, the K-product is formed by the easy transfer of the chloride anion to the carbonyl carbon *via*K-Ts2, which demands an energy of 13.5 kcal mol^−1^ (1.6 kcal mol^−1^ less stable than A-Ts2). Note that 2.09 Å is the breaking B–Cl distance, while 2.17 Å is the forming C–Cl distance.

**Fig. 2:**
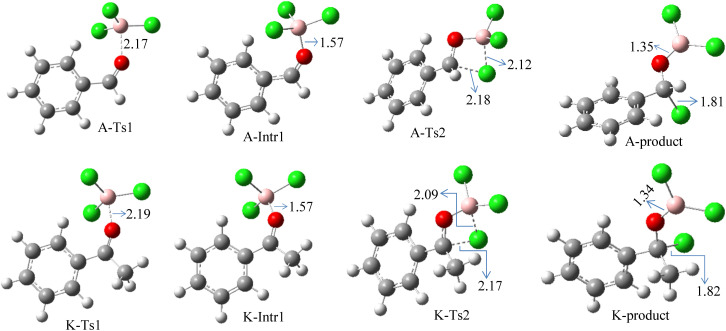
Geometric structures of intermediates and transition states in the chloroboration of benzaldehyde and acetophenone (distances are in Å, carbon: gray, hydrogen: white, oxygen: red, boron: pink, chlorine: green).

According to DFT results displayed in Fig. 1, aldehydes exhibited a higher reactivity than ketones owing to the steric hindrance effect and ketones' reduced electrophilicity. Fig. 2 shows the structure of the transition states and optimized intermediates during the chloroboration of aldehydes and ketones.

**Fig. 3:**
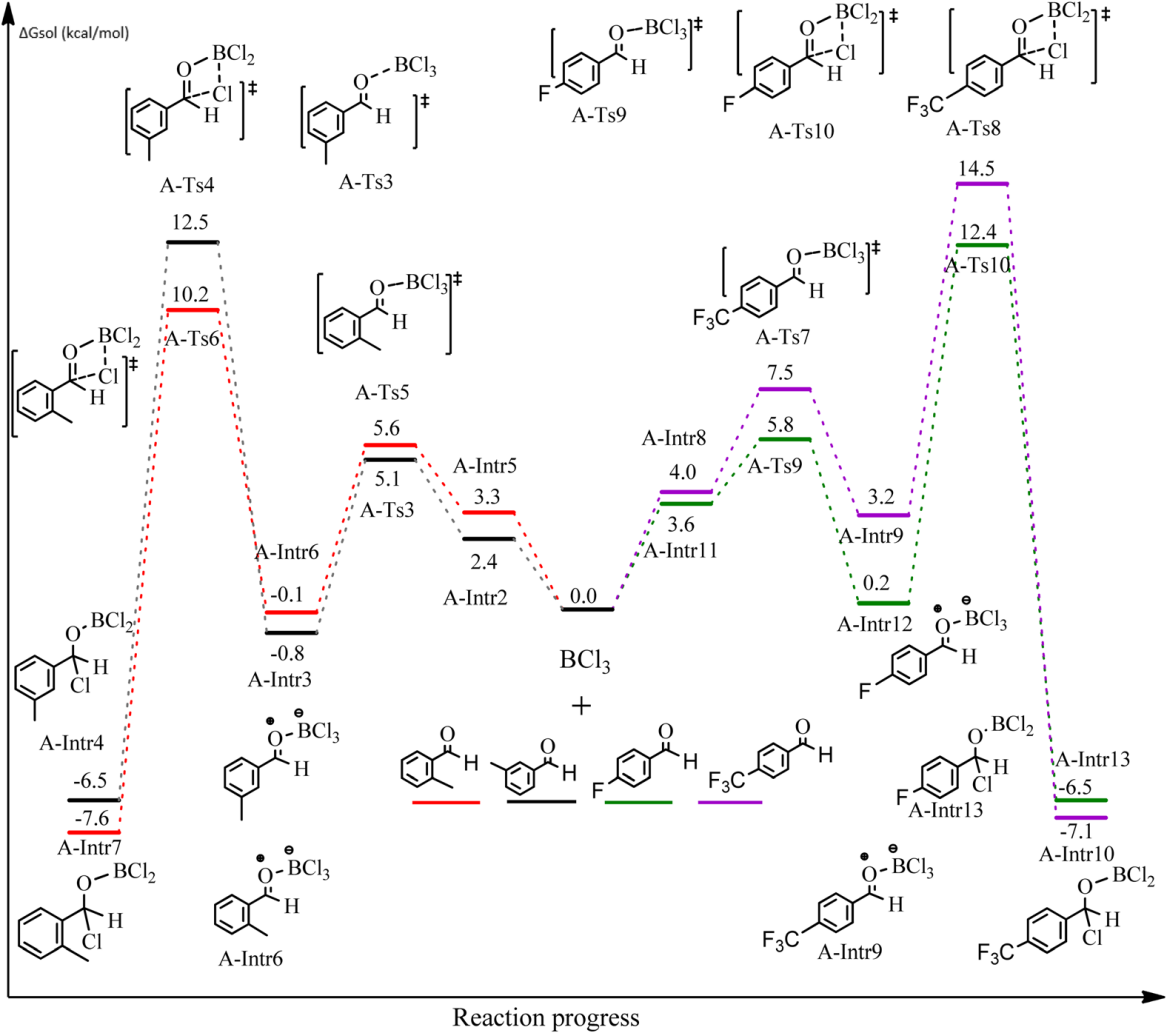
DFT-computed free energies for chloroboration reaction of substituted benzaldehyde bearing *m*-CH_3_, *o*-CH_3_, *p*-CF_3_, and *p*-F.

**Fig. 4:**
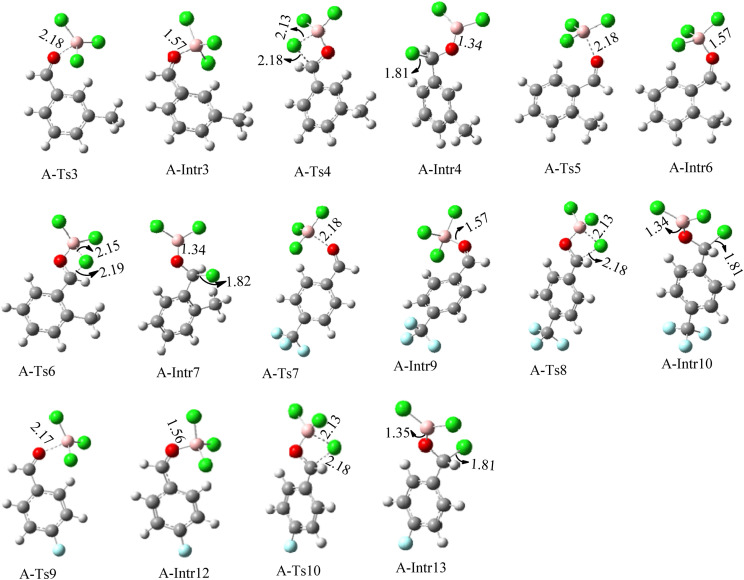
Geometric structures of intermediates and transition states in the chloroboration of substituted benzaldehyde bearing *m*-CH_3_, *o*-CH_3_, *p*-CF_3_ and *p*-F (distances are given in Å).

**Fig. 5:**
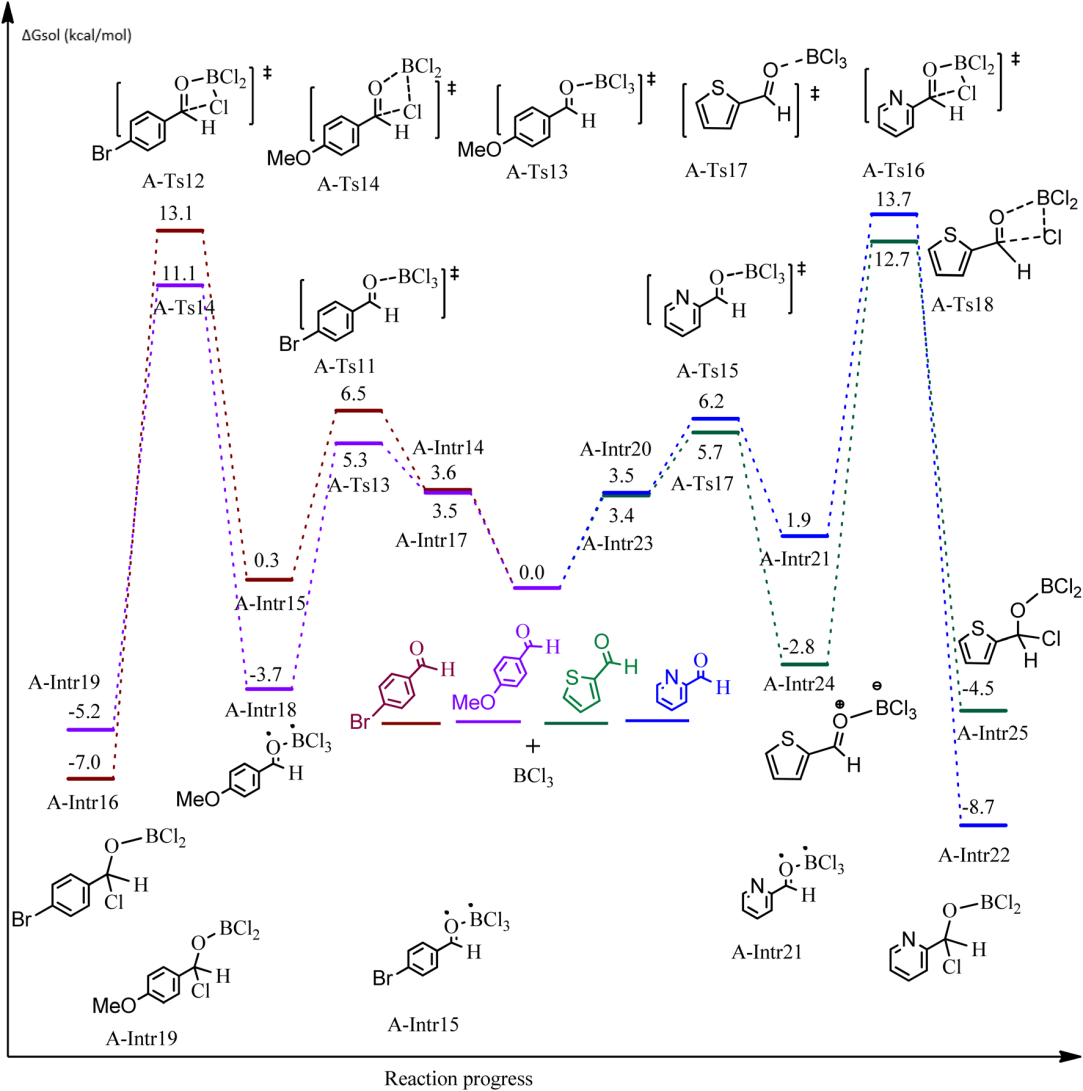
DFT-computed free energies for the chloroboration reaction of substituted benzaldehyde bearing *p*-Br and *p*-OMe, picolinbenzaldehyde and thiophen-2-carbaldehyde.

**Fig. 6:**
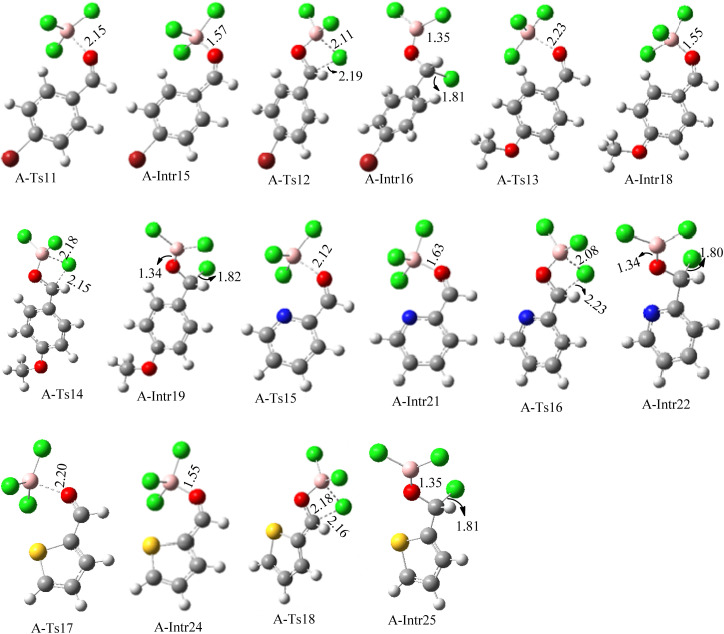
Geometric structures for intermediates and transition states in the chloroboration of substitute benzaldehyde bearing *p*-Br, *p*-OMe, picolinbenzaldehyde and thiophen-2-carbaldehyde (distances are given in Å).

After we performed DFT calculation for the model substrate, we looked at the extent of the reaction when specific substituted benzaldehydes bearing methyl, methoxy, halogens, and heterocyclic derivatives were functionalized with either electron-withdrawing or electron-donating groups Table 1. Fig. 3 presents DFT results for the BCl_3_-induced chloroboration reaction of substituted benzaldehydes bearing *m*-CH_3_, *o*-CH_3_, *p*-CF_3_, and *p*-F. In contrast to the parent model substrate benzaldehyde (Fig. 1), the chloroboration of aldehydes substituted with *o*-CH_3_ and *p*-CF_3_ proceeded with lower activation energy, yielding more stable boronate ester products (Fig. 3). The reaction proceeded with somewhat greater activation energy to afford the predicted boronate esters when the substituent groups are *m*-CH_3_ and *p*-F when compared with the parent substrate benzaldehyde. Fig. 5 shows the DFT results for the BCl_3_-induced chloroboration reaction of substituted benzaldehydes bearing *p*-Br and *p*-OMe as well as picolinbenzaldehyde and thiophen-2-carbaldehyde as a substrate. *p*-Br and *p*-OMe substituted benzaldehyde and thiophen-2-carbaldehyde demands a higher energy barrier than the model reactant. Picolinbenzaldehyde consumes energy almost equal to that consumed by benzaldehyde. Fig. 4 and 6 show the geometric structures for intermediates and transition states in the chloroboration of substituted benzaldehyde.

**Table 1:** DFT-computed free energies (kcal mol^−1^) for B–O bond formation and 1,3-Cl migration steps in the chloroboration reaction of model and substituted aldehyde substrates

Substrate	B–O bond formation	1,3-Cl migration	Substrate	B–O bond formation	1,3-Cl migration
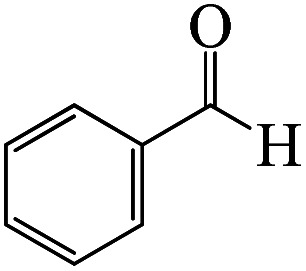	2.1	11.9	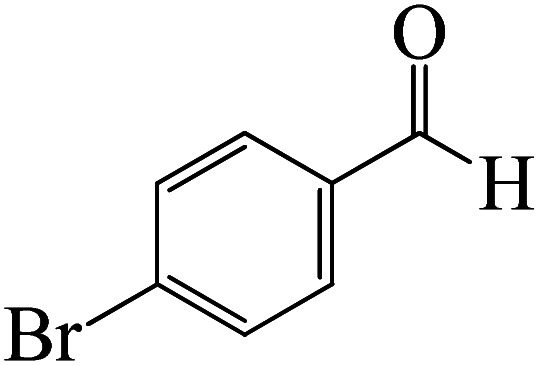	2.9	12.8
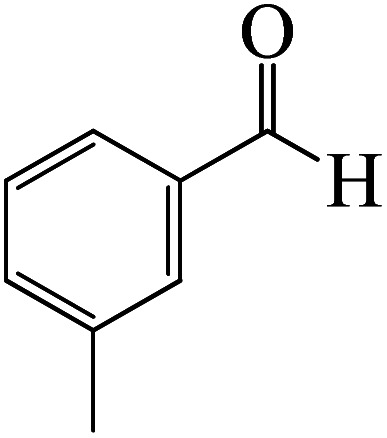	2.7	13.3	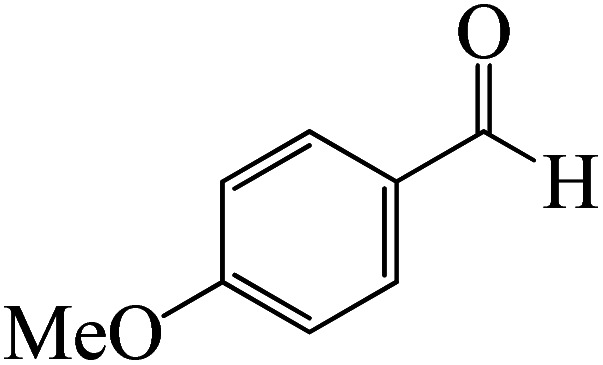	1.8	14.8
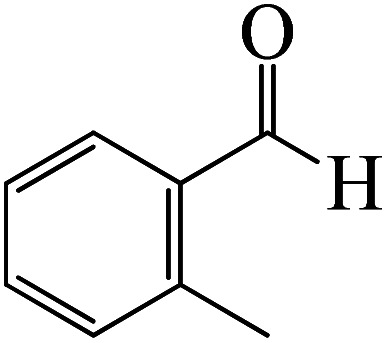	2.3	10.3	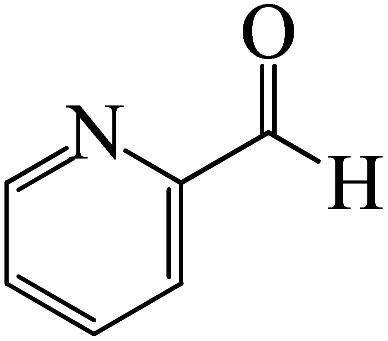	2.7	11.8
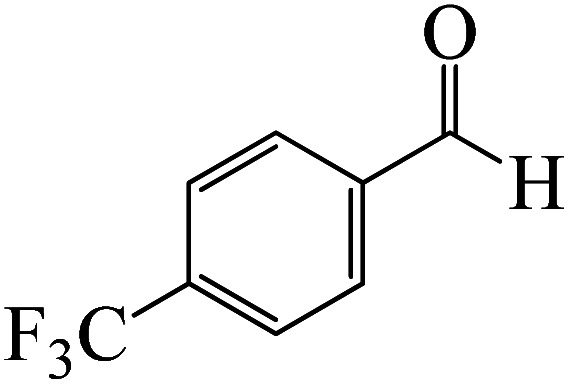	3.5	11.3	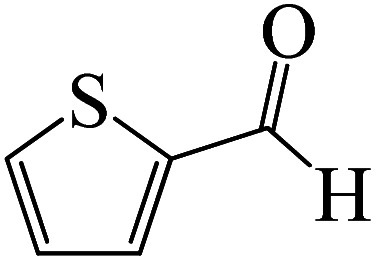	2.3	15.5
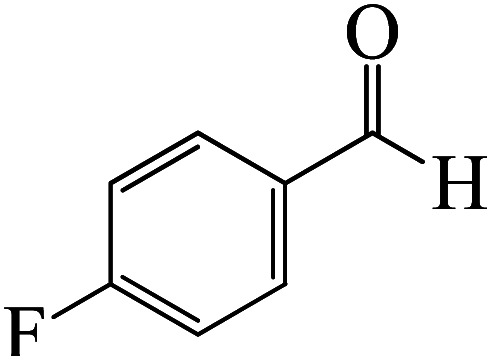	2.2	12.2			

The production of the coordinated complex A-Intr2 between the C=O group of the reactant (3-methylbenzaldehyde) and BCl_3_ is found to be 2.4 kcal mol^−1^ based on the calculation results given in Fig. 3. A-Intr2 is then converted *via*A-Ts3 into the zwitterion intermediate A-Intr3, which has a lower activation energy of 2.7 kcal mol^−1^. Notably, 13.3 kcal mol^−1^ (1.4 kcal mol^−1^ greater than that of the model reactant) is needed for the four-membered ring transition state A-Ts4 to form the borylated intermediate A-Intr4. The coordinated complex A-Intr5 is determined to be 3.3 kcal mol^−1^ when CH_3_ is in *ortho*-position (2-methylbenzaldehyde), and A-Ts5 forms A-Intr6 (requiring 2.3 kcal mol^−1^). A-Ts6 converts A-Intr6 into A-Intr7 at 10.3 kcal mol^−1^, which is 1.6 kcal mol^−1^ more stable than the model reactant and 3.0 kcal mol^−1^ from 3-methylbenzaldehyde. By increasing the electron density in the benzene ring, the CH_3_ group, an activating group, promotes the reaction progress.

The formation of the coordinated complex A-Intr8 between the C=O group of 4-(trifluoromethyl)benzaldehyde and BCl_3_ is reported to be 4.0 kcal mol^−1^ based on the computation results displayed in Fig. 3. Next, by converting A-Intr8*via*A-Ts7 (3.5 kcal mol^−1^), the zwitterion intermediate A-Intr9 is produced; 11.3 kcal mol^−1^ is required for the four-membered ring transition state A-Ts8 to produce the borylated intermediate A-Intr10. The coordinated complex A-Intr11 is determined to be 3.6 kcal mol^−1^ when the substituent is *p*-F. This is followed by the production of A-Intr12*via*A-Ts9, which requires 2.2 kcal mol^−1^. A-Ts10 converts A-Intr12 into A-Intr13 at a cost of 12.2 kcal mol^−1^. This indicates that compared to 4-(fluoro)benzaldehyde, 4-(trifluoromethyl)benzaldehyde is more reactive because compared to F atoms, CF_3_ is a more potent electron-withdrawing group. The structure of the optimal intermediates and transition states during the chloroboration of replacement benzaldehyde is shown in Fig. 4.

ased on the computation findings shown in Fig. 5, the synthesis of the coordinated complex A-Intr14 between the C=O group of the reactant (4-bromobenzaldehyde) and BCl_3_ is found to be 3.6 kcal mol^−1^. The zwitterion intermediate A-Intr15, which has lower activation energy of 2.9 kcal mol^−1^, is subsequently created by converting A-Intr14*via*A-Ts11. The borylated intermediate A-Intr16 is obtained by converting the four-membered ring transition state A-Ts12 to 12.8 kcal mol^−1^. When *p*-OMe is the substituent, A-Intr17 is formed with 3.5 kcal mol^−1^ of energy, while A-Intr18 through A-Ts13 is formed with 1.8 kcal mol^−1^. As a result, A-Ts14 forms A-Intr19, requiring 14.8 kcal mol^−1^ of energy, which makes it 2.9 kcal mol^−1^ less stable than the model substrate.

The synthesis of the coordinated complex A-Intr20 between C=O group picolinbenzaldehyde and BCl_3_ is found to be 3.5 kcal mol^−1^, based on the computation results displayed in Fig. 5. Next, using A-Ts15 with 2.7 kcal mol^−1^, A-Intr20 is converted into the zwitterion intermediate A-Intr21. The four-membered ring transition state A-Ts16 requires 11.8 kcal mol^−1^ free energy to yield the borylated intermediate A-Intr22. A-Intr23 (3.4 kcal mol^−1^) is produced when thiophen-2-carbaldehyde is used as the substrate in a reaction with BCl_3_. A-Ts17 (2.3 kcal mol^−1^) then converts this product into A-Intr24. A-Intr24 is converted into A-Intr25*via*A-Ts18 (15.5 kcal mol^−1^, 3.6 kcal mol^−1^ less stable than the model reactant).

**Fig. 7:**
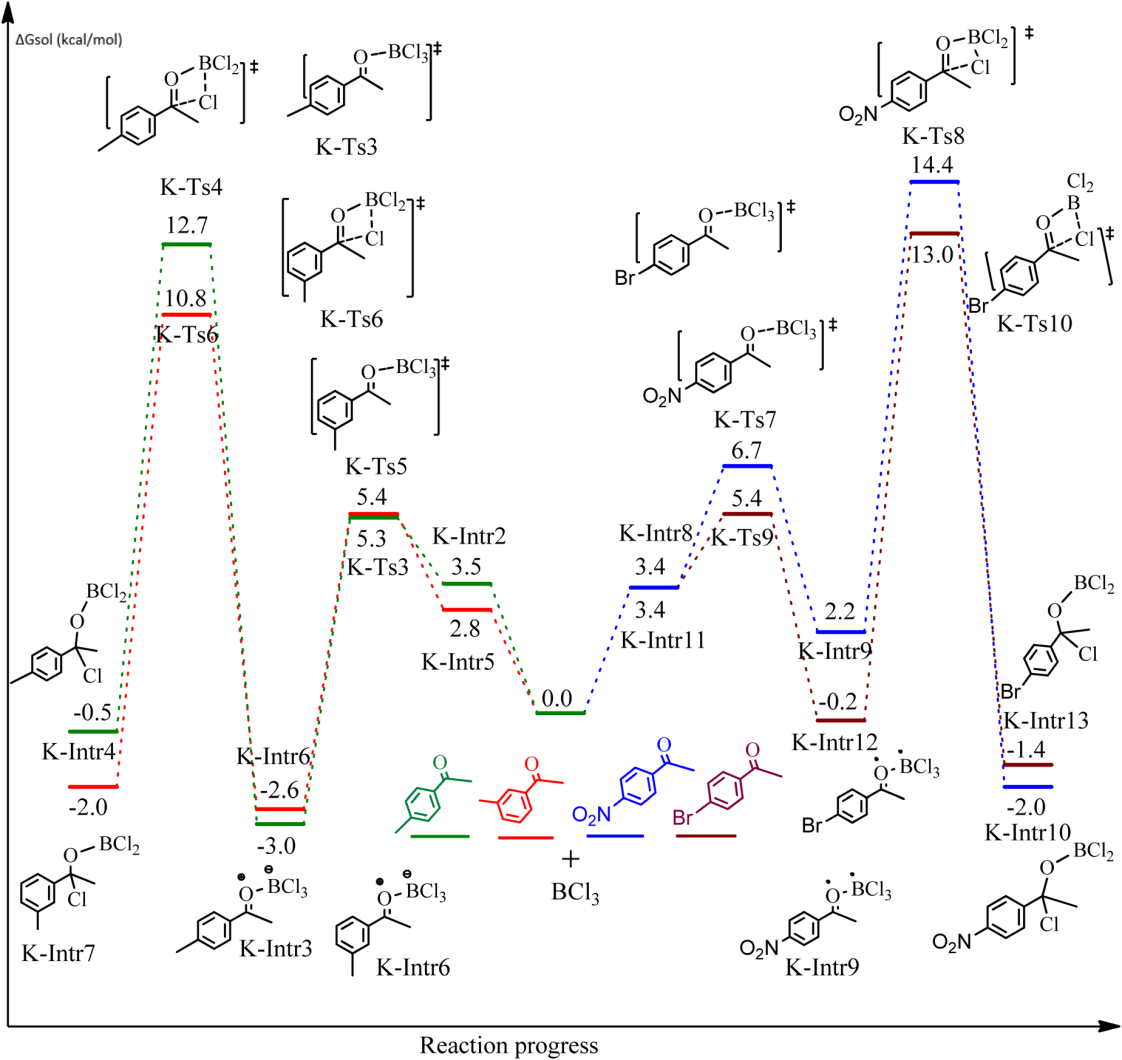
DFT-computed free energies for the chloroboration reaction of substituted acetophenone bearing *p*-CH_3_, *m*-CH_3_, *p*-NO_2_ and *p*-Br.

**Fig. 8:**
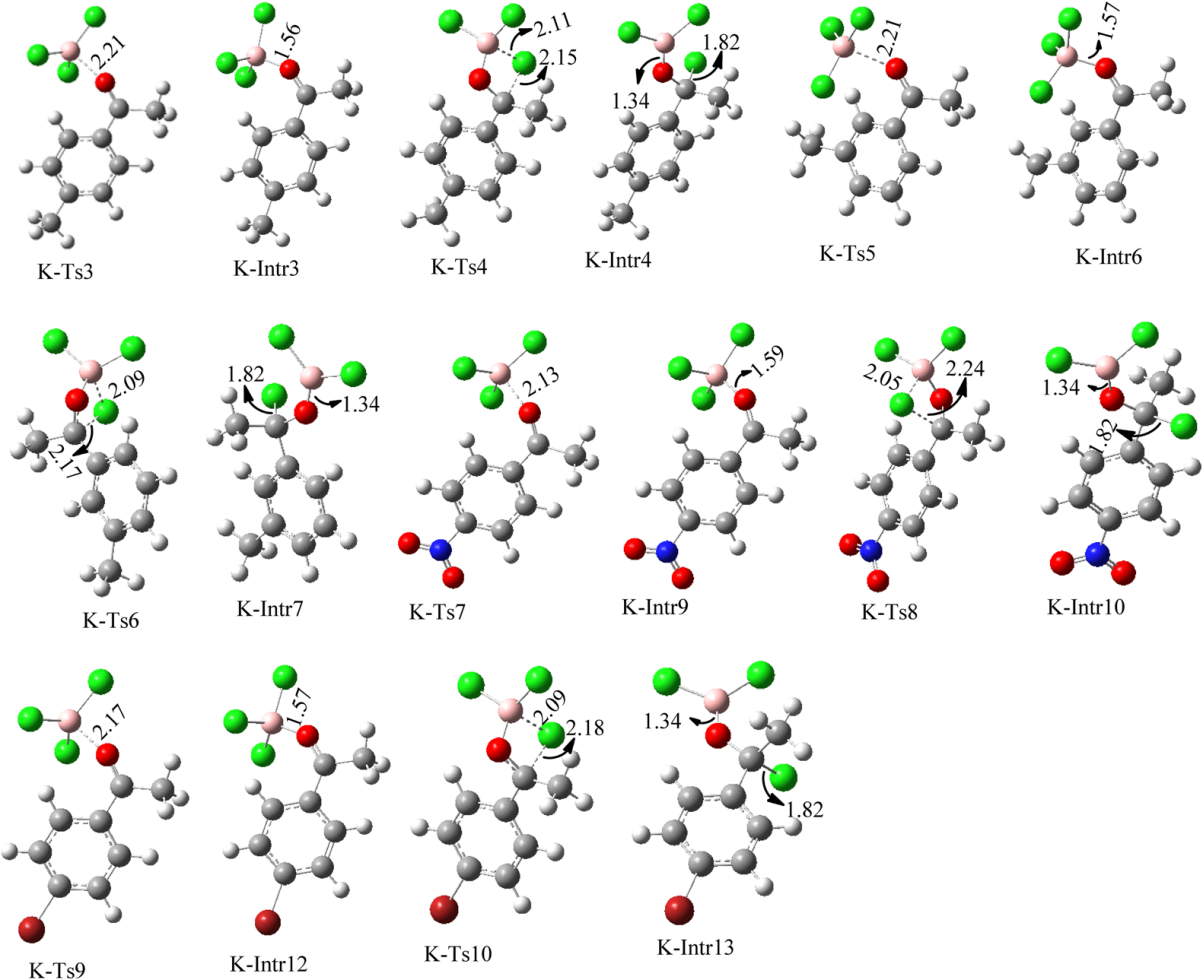
Geometric structures of intermediates and transition states in the chloroboration of substituted acetophenone bearing *p*-CH_3_, *o*-CH_3_, *p*-NO_2_ and *p*-Br (distances are given in Å).

**Fig. 9:**
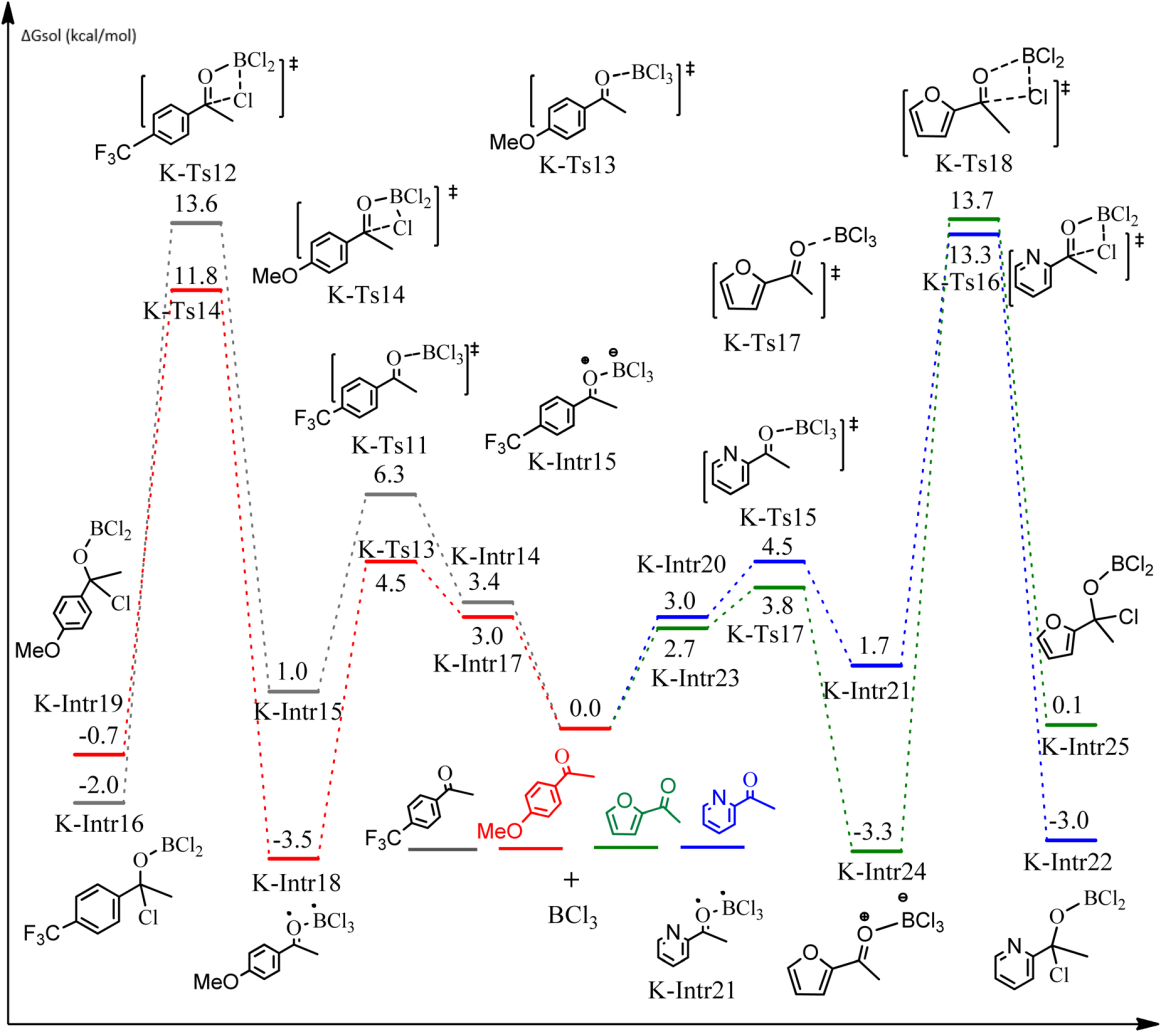
DFT-computed free energies for the chloroboration reaction of substituted acetophenone bearing *p*-CF_3_, *p*-OMe, 1-(pyridin-2-yl)ethanone and 1-(furan-2-yl)ethanone.

**Fig. 10:**
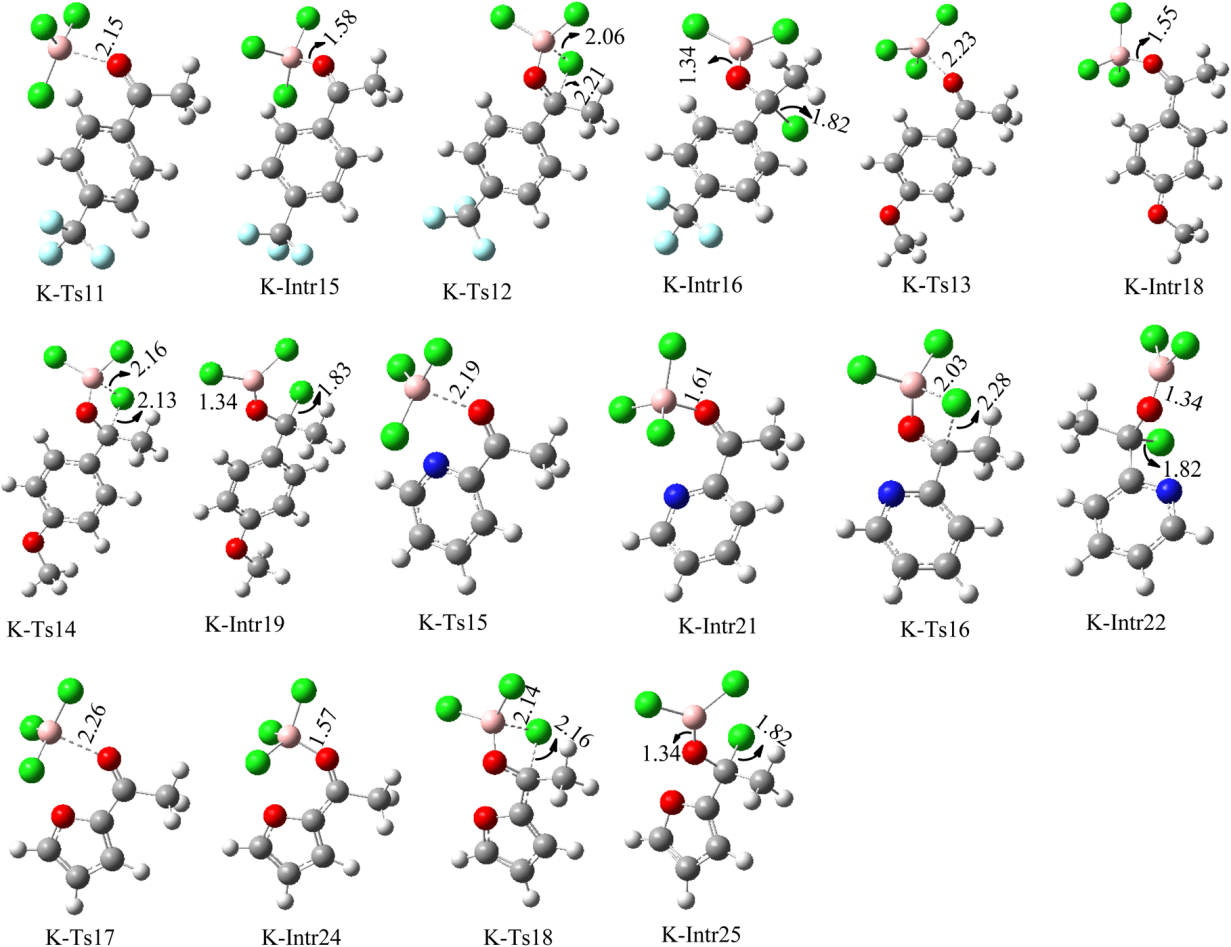
Geometric structures of intermediates and transition states in the chloroboration of substituted acetophenone bearing *p*-CF_3_, *p*-OMe, 1-(pyridin-2-yl)ethanone and 1-(furan-2-yl)ethanone (distances are given in Å).

Following a computational study on the chloroboration of substituted benzaldehyde, we performed DFT calculations to better understand the scope of BCl_3_-promoted chloroboration of ketones in a catalyst-free manner when specific substituted acetophenones containing methyl, methoxy, halogens, nitro, and heterocyclic derivatives were functionalized with either electron-donating or electron-withdrawing groups Table 2. Fig. 7 and 9 show DFT-computed free energies for the chloroboration reactions of substituted acetophenone. Fig. 8 and 10 show the geometric structures for intermediates and transition states in the chloroboration of substituted acetophenone.

**Table 2:** DFT-computed free energies (kcal mol^−1^) for B–O bond formation and 1,3-Cl migration steps in the chloroboration reaction of model and substituted ketone substrates

Substrate	B–O bond formation	1,3-Cl migration	Substrate	B–O bond formation	1,3-Cl migration
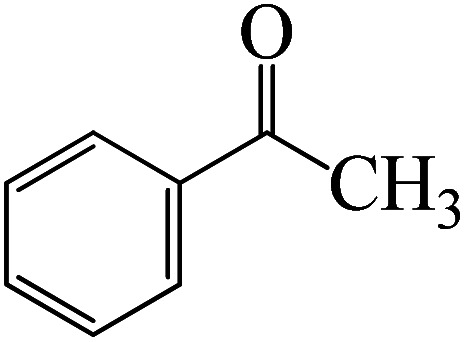	1.8	13.5	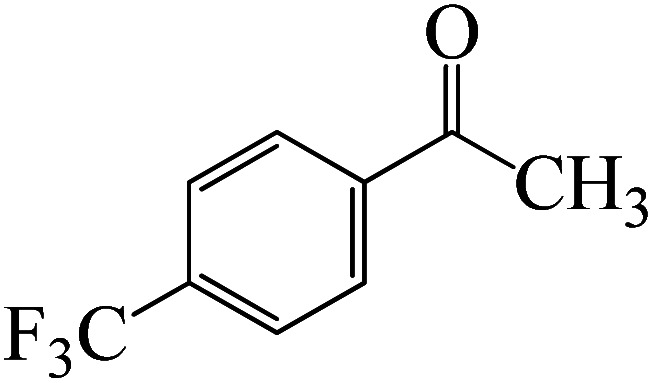	2.9	12.6
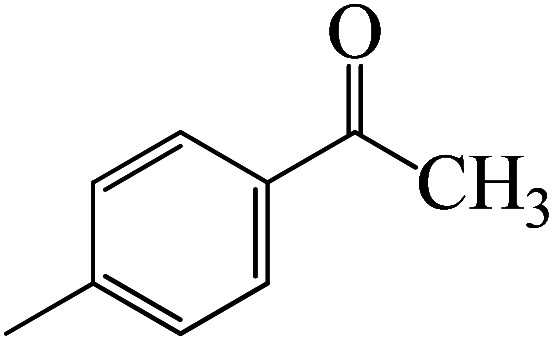	1.8	15.7	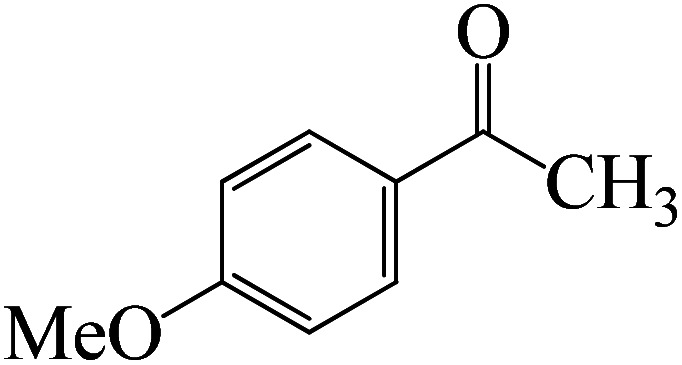	1.5	15.3
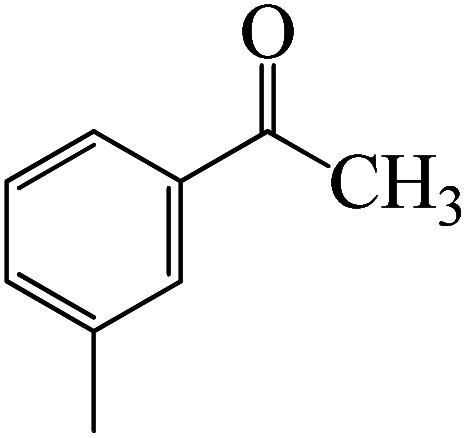	2.6	13.4	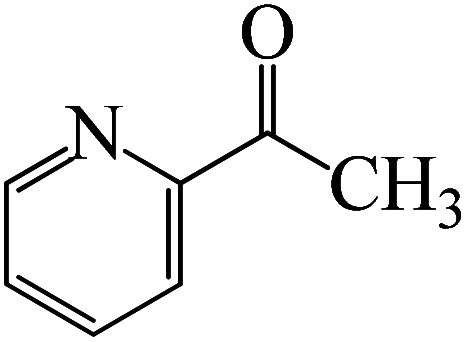	1.5	11.6
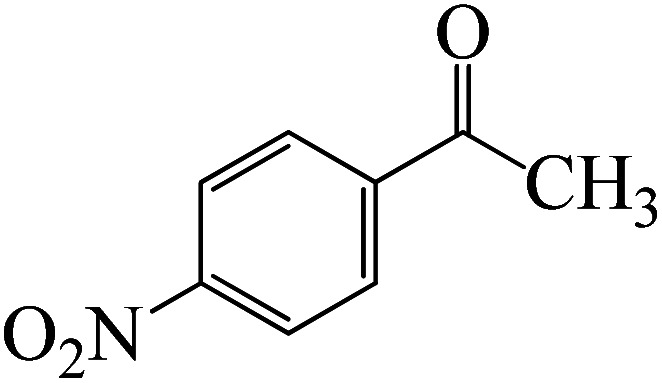	3.3	12.2	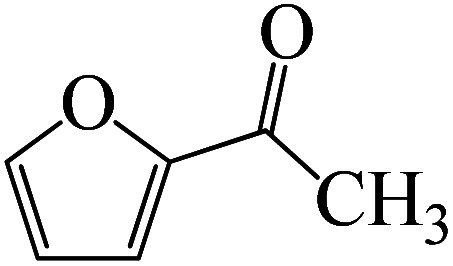	1.1	17.0
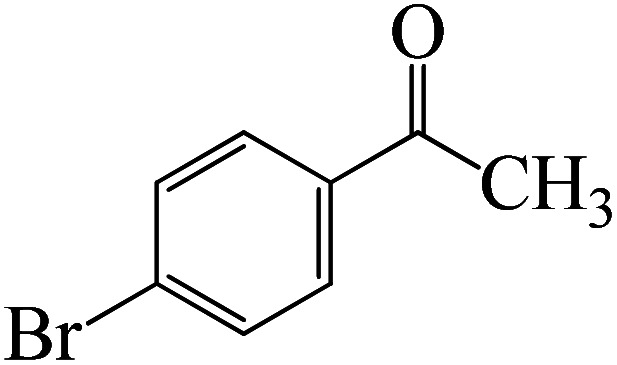	2.0	13.2			

Complexation of BCl_3_ with 1-(*p*-tolyl)ethanone, which occurs kinetically at 3.5 kcal mol^−1^ to produce K-Intr2, is the first step. K-Intr3 is then generated through the transition state K-Ts3, which has a lower energy barrier of 1.8 kcal mol^−1^. After K-Intr3 (2.4 kcal mol^−1^) is created, K-Ts4 (15.7 kcal mol^−1^) facilitates the easy transfer of the chloride anion to the carbonyl carbon to generate K-Intr4. It is less stable by 2.2 kcal mol^−1^ compared to the model substrate. The coordinated complex A-Intr5 forms with a reduced activation energy of 2.8 kcal mol^−1^ when the substrate is 1-(*m*-tolyl)ethanone. With a lower energy barrier of 2.6 kcal mol^−1^, the previous transition state, K-Ts5, is more kinetically advantageous to produce K-Intr6. After K-Intr6 is produced, K-Ts6, which is 13.4 kcal mol^−1^, may be used to transfer the chloride anion to the carbonyl carbon with ease, yielding the desired product, K-Intr7. In comparison with K-Ts4 of 1-(*p*-tolyl)ethanone, K-Ts6 of 1-(*m*-tolyl)ethanone is stable by 2.3 kcal mol^−1^.

The formation of the complex intermediate K-Intr8 (3.4 kcal mol^−1^) occurs when 1-(4-nitrophenyl)ethanone is the substrate. K-Intr9 is generated through the transition state K-Ts7, which has a lower energy barrier of 3.3 kcal mol^−1^. After K-Intr9 (2.2 kcal mol^−1^) is created, K-Ts8, which is 12.2 kcal mol^−1^ (1.3 kcal mol^−1^ stable than the model substrate), facilitates the easy transfer of the chloride anion to the carbonyl carbon, generating K-Intr10. The coordinated complex A-Intr11 is generated with an activation energy of 3.4 kcal mol^−1^ when the substrate is 1-(4-bromophenyl)ethanone. With a lower energy barrier of 2.0 kcal mol^−1^, the previous transition state, K-Ts9, is more kinetically advantageous to produce K-Intr12. K-Ts10, which is 13.2 kcal mol^−1^, makes it simple to move the chloride anion to the carbonyl carbon once K-Intr12 is formed. K-Intr13 is the final product of this step.

As shown in Fig. 9, the C=O moiety of the 1-(4-(trifluoromethyl)phenyl)ethanone reactant molecule combines with BCl_3_. K-Intr14 is kinetically advantageous since K-Ts11 has a lower energy barrier of 2.9 kcal mol^−1^. K-Intr16 is produced by the rapid transfer of the chloride anion to the carbonyl carbon *via*K-Ts12 (12.6 kcal mol^−1^), which occurs after K-Intr15 (1.0 kcal mol^−1^) is formed. When OMe is a substituent of acetophenone at *para*-position, 1-(4-methoxyphenyl)ethanone forms the complex K-Intr17 with BCl_3_ by demanding 3.0 kcal mol^−1^. This is followed by the synthesis of K-Intr18 through K-Ts13, which requires 1.5 kcal mol^−1^. Finally, using K-Ts14, K-Intro19 is generated with an activation energy of 15.3 kcal mol^−1^ (1.8 kcal mol^−1^ higher than that of the model reactant) of energy.

The C=O moiety of 1-(pyridin-2-yl)ethanone reacts with BCl_3_ to generate K-intr20 (3.0 kcal mol^−1^) as the reactant. As can be observed in Fig. 9, K-Ts15, the preceding transition state, has a lower energy barrier of 1.5 kcal mol^−1^, making K-Intr21 kinetically favorable. When the chloride anion is shifted from K-Intr21 to the carbonyl carbon *via*K-Ts16 (11.6 kcal mol^−1^, 1.9 kcal mol^−1^ stable than the model reactant), K-Intr22 is formed. While 1-(furan-2-yl)ethanone serves as the substrate, K-Intr23 is created at a rate of 2.7 kcal mol^−1^. For K-Ts17 to convert K-Intr23 into K-Intr24, 1.1 kcal mol^−1^ is required. Finally, K-Ts18, which requires 17.0 kcal mol^−1^, transfers K-Intr24 to K-Intr24. K-Ts18 makes 1-(furan-2-yl)ethanone less stable than the model substrate by 3.5 kcal mol^−1^.

## Conclusions

In conclusion, DFT calculations were used to carry out a mechanistic analysis of catalyst-free interactions between carbonyl compounds and BCl_3_. We suggest that the interaction between the C=O bond and BCl_3_ activates the chloroboration of carbonyl compounds. The mechanism yields an overall free energy barrier of <20 kcal mol^−1^, which is in good agreement with the observation that reactions take place at room temperature, according to the DFT result. We reported that BCl_3_ facilitated catalyst-free chloroboration reactions of carbonyl compounds, supporting the universality of the finding. Therefore, our computations offer mechanistic insights into the crucial reactions involved in the synthesis of chemicals derived from organoborane derivatives, and they could be beneficial in the development and execution of novel reactions of this type.

## Data availability

The authors confirm that the data supporting the findings of this study are available within the article and its ESI material.†

## Conflicts of interest

There are no conflicts to declare.

## Supplementary Material

RA-015-D4RA06893A-s001
