# Supplementary material for: Computational study on catalyst-free BCl3-promoted chloroboration of carbonyl compounds
Source: RSC Adv. 2025 Jan 28;15(4):2862–73. doi: 10.1039/d4ra06893a (PMC11774190; doi:10.1039/d4ra06893a)
Supplement: RA-015-D4RA06893A-s001 [file RA-015-D4RA06893A-s001.pdf]

Supplementary Information (SI) for RSC Advances.
This journal is © The Royal Society of Chemistry 2025

Journal Name: RSC Advances

Supplementary Material

Computational study on catalyst-free BCl_3 promoted chloroboration of carbonyl compounds

Abera Tadie Derese*[a], Mengistu Gemech Menkir[b], Mihret Kindie Wolie[c], and Desalegn Addis Yemam[d]

[a,d]Department of Chemistry, Debre Tabor University, Ethiopia

[b]Department of Chemistry, Bahir Dar University, Ethiopia

[c]Department of Chemistry, Debark University, Ethiopia

*corresponding author email: aberatadie@gmail.com

Cartesian coordinates (Å) for optimized structures of model substrates for both aldehydes and ketones in toluene at M062X/6-311++G(d,p) level with PCM model. The coordinates of the optimized structures in gas phase are not included.

A-Ts1

Center Number	Atomic Number	Atomic Type	Coordinates (Angstroms) X	Y	Z
1	6	0	-2.506166	2.586841	0.593865
2	6	0	-1.341787	2.775833	-0.147795
3	6	0	-0.192609	3.229659	0.487198
4	6	0	-0.212926	3.508773	1.857545
5	6	0	-1.381489	3.308737	2.601460
6	6	0	-2.524922	2.846294	1.965165
7	1	0	-3.405407	2.228120	0.102063
8	1	0	-1.331599	2.566319	-1.212250
9	1	0	0.725659	3.376920	-0.076646
10	1	0	-1.378549	3.500267	3.668910
11	1	0	-3.433728	2.684902	2.535547
12	6	0	1.027376	3.994852	2.476206
13	8	0	1.159851	4.434502	3.605659
14	5	0	0.113890	5.474694	5.193498
15	17	0	1.535678	6.358017	5.735448

16	17	0	-1.077788	6.322535	4.195107
17	17	0	-0.413647	4.064551	6.125497
18	1	0	1.923267	3.943514	1.830410

A-Intr1

Center Number	Atomic Number	Atomic Type	Coordinates (Angstroms) X	Y	Z
1	6	0	-2.113116	-2.038323	-2.966902
2	6	0	-0.872263	-1.936553	-3.595272
3	6	0	0.174863	-1.324218	-2.927810
4	6	0	-0.005324	-0.808440	-1.627987
5	6	0	-1.263355	-0.916640	-1.003049
6	6	0	-2.304804	-1.531139	-1.681011
7	1	0	-2.938974	-2.518079	-3.483461
8	1	0	-0.727652	-2.332911	-4.594159
9	1	0	1.146562	-1.238631	-3.407384
10	1	0	-1.423414	-0.527096	-0.008155
11	1	0	-3.274485	-1.616617	-1.203039
12	6	0	1.182792	-0.204277	-1.071182
13	8	0	1.419931	0.341264	0.021599
14	5	0	0.646660	0.675556	1.342712

15	17	0	1.892274	1.495198	2.378237
16	17	0	-0.730015	1.798382	0.869972
17	17	0	0.094148	-0.931238	2.044098
18	1	0	2.057648	-0.223404	-1.730603

A-Ts2

Center Number	Atomic Number	Atomic Type	Coordinates (Angstroms) X	Y	Z
1	6	0	-2.005855	-2.439896	-3.105570
2	6	0	-0.793897	-2.071147	-3.689014
3	6	0	0.074596	-1.237990	-2.997965
4	6	0	-0.275124	-0.779068	-1.723511
5	6	0	-1.494067	-1.143363	-1.139929
6	6	0	-2.355793	-1.979331	-1.835248
7	1	0	-2.685233	-3.091384	-3.646823
8	1	0	-0.530718	-2.433538	-4.676972
9	1	0	1.021377	-0.936999	-3.439320
10	1	0	-1.741170	-0.765899	-0.152843
11	1	0	-3.302099	-2.272428	-1.392904
12	6	0	0.648948	0.084048	-1.003500
13	8	0	0.482013	0.226867	0.299212

14	5	0	0.777409	1.619107	0.607280
15	17	0	-0.344687	2.336933	1.788620
16	17	0	2.527448	1.998611	0.781881
17	17	0	0.226174	2.193448	-1.359225
18	1	0	1.664615	0.183078	-1.393923

A-product

Center Number	Atomic Number	Atomic Type	Coordinates (Angstroms) X	Y	Z
1	6	0	-1.967403	-3.119698	-2.339198
2	6	0	-0.712721	-2.790242	-2.844952
3	6	0	0.026396	-1.769927	-2.255341
4	6	0	-0.489583	-1.078253	-1.161576
5	6	0	-1.746413	-1.404866	-0.654672
6	6	0	-2.481272	-2.427866	-1.244447
7	1	0	-2.544084	-3.917556	-2.796779
8	1	0	-0.307424	-3.329120	-3.695453
9	1	0	1.005552	-1.506626	-2.648214
10	1	0	-2.137023	-0.862515	0.200345
11	1	0	-3.458349	-2.685638	-0.848054
12	6	0	0.313931	0.040643	-0.567306

13	8	0	0.030883	0.162058	0.801772
14	5	0	0.848787	0.739858	1.700681
15	17	0	0.286245	0.844888	3.357012
16	17	0	2.443086	1.353507	1.271396
17	17	0	-0.087668	1.613266	-1.373298
18	1	0	1.384569	-0.094119	-0.731058

K-Ts1

Center	Atomic	Atomic	Coordinates (Angstroms)		
Number	Number	Type	X	Y	Z
1	6	0	-2.555005	2.565649	0.496154
2	6	0	-1.374560	2.631856	-0.241249
3	6	0	-0.201663	3.062401	0.367048
4	6	0	-0.208188	3.447352	1.712613
5	6	0	-1.396347	3.375603	2.447698
6	6	0	-2.564212	2.932285	1.840769
7	1	0	-3.469916	2.223260	0.022063
8	1	0	-1.368318	2.345881	-1.288083
9	1	0	0.715271	3.109221	-0.212408
10	1	0	-1.389399	3.655306	3.496207
11	1	0	-3.482575	2.874182	2.415837

12	6	0	1.033069	3.913187	2.384517
13	8	0	1.010538	4.514721	3.450985
14	6	0	2.367534	3.625415	1.739112
15	1	0	2.446815	2.575964	1.442975
16	1	0	2.478064	4.237029	0.836601
17	1	0	3.159834	3.884406	2.440702
18	5	0	-0.096938	6.053345	4.547632
19	17	0	1.348469	6.968854	4.960271
20	17	0	-1.117008	6.634724	3.225885
21	17	0	-0.815111	5.006213	5.778509

K-Intr1

Center Number	Atomic Number	Atomic Type	Coordinates (Angstroms) X	Y	Z
1	6	0	-2.246641	-2.186794	-2.574102
2	6	0	-1.051280	-2.044142	-3.279577
3	6	0	0.074937	-1.561841	-2.632561
4	6	0	0.002490	-1.179642	-1.281548
5	6	0	-1.203467	-1.323602	-0.579631
6	6	0	-2.318100	-1.838417	-1.227565
7	1	0	-3.125836	-2.576665	-3.078033

8	1	0	-0.999915	-2.314478	-4.328764
9	1	0	1.003882	-1.455396	-3.183595
10	1	0	-1.256143	-1.061045	0.470603
11	1	0	-3.246374	-1.959928	-0.680066
12	6	0	1.215087	-0.700552	-0.619874
13	8	0	1.253989	0.034813	0.390794
14	6	0	2.562449	-1.104982	-1.142549
15	1	0	2.574449	-2.147132	-1.465074
16	1	0	2.800642	-0.474355	-2.008226
17	1	0	3.312415	-0.923837	-0.373327
18	5	0	0.340736	1.048564	1.167234
19	17	0	1.535498	2.281519	1.780288
20	17	0	-0.863225	1.779407	-0.004960
21	17	0	-0.436300	0.086666	2.527077

K-Ts2

Center	Atomic	Atomic	Coordinates (Angstroms)		
Number	Number	Type	X	Y	Z
1	6	0	-2.273559	-2.301830	-2.573352
2	6	0	-1.061286	-2.303941	-3.260844
3	6	0	0.060011	-1.714292	-2.691691

4	6	0	-0.028635	-1.125360	-1.425034
5	6	0	-1.248976	-1.123273	-0.737611
6	6	0	-2.366444	-1.711237	-1.313934
7	1	0	-3.149153	-2.761253	-3.021639
8	1	0	-0.989651	-2.763865	-4.240891
9	1	0	0.998383	-1.717245	-3.235781
10	1	0	-1.304708	-0.657064	0.240864
11	1	0	-3.311517	-1.708781	-0.781113
12	6	0	1.146623	-0.503757	-0.783429
13	8	0	1.037017	-0.323518	0.539890
14	6	0	2.532652	-0.792126	-1.282335
15	1	0	2.734634	-1.845254	-1.049653
16	1	0	2.629732	-0.634486	-2.356006
17	1	0	3.252564	-0.170891	-0.748635
18	5	0	1.159550	1.083162	0.845434
19	17	0	2.780341	1.622703	1.421277
20	17	0	0.985635	1.626475	-1.168849
21	17	0	-0.250440	1.741827	1.743774

K-product

Center	Atomic	Atomic	Coordinates (Angstroms)		
Number	Number	Type	X	Y	Z

1	6	0	-2.655643	-2.580297	-2.211463
2	6	0	-1.544371	-2.267782	-2.993151
3	6	0	-0.520991	-1.489739	-2.468811
4	6	0	-0.597024	-1.015409	-1.156727
5	6	0	-1.707391	-1.328288	-0.375574
6	6	0	-2.733642	-2.108105	-0.906663
7	1	0	-3.457323	-3.186169	-2.622115
8	1	0	-1.477684	-2.625839	-4.015626
9	1	0	0.332797	-1.228781	-3.088283
10	1	0	-1.772546	-0.960441	0.641590
11	1	0	-3.597352	-2.344028	-0.292916
12	6	0	0.565969	-0.223581	-0.583057
13	8	0	0.168890	0.261692	0.679227
14	6	0	1.822651	-1.073438	-0.464196
15	1	0	1.613657	-1.893861	0.228866
16	1	0	2.077442	-1.493654	-1.438606
17	1	0	2.664673	-0.483242	-0.099077
18	5	0	0.773112	1.068038	1.563390
19	17	0	2.354444	1.809309	1.353040
20	17	0	0.909440	1.192441	-1.678106

21	17	0	-0.108403	1.374342	3.057772
